# Genomic and clinical characterisation of multidrug-resistant carbapenemase-producing ST231 and ST16 *Klebsiella pneumoniae* isolates colonising patients at Siriraj hospital, Bangkok, Thailand from 2015 to 2017

**DOI:** 10.1186/s12879-021-05790-9

**Published:** 2021-02-04

**Authors:** Adhiratha Boonyasiri, Elita Jauneikaite, Lauren M. Brinkac, Chris Greco, Kanokorn Lerdlamyong, Teerawit Tangkoskul, Kevin Nguyen, Visanu Thamlikitkul, Derrick E. Fouts

**Affiliations:** 1 Department of Research and Development, Faculty of Medicine, Siriraj Hospital, Mahidol University, Bangkok, Thailand; 2 NIHR Health Protection Research Unit in Healthcare Associated Infections and Antimicrobial Resistance, Imperial College, London, UK; 3 Department of Infectious Disease Epidemiology, School of Public Health, Imperial College, London, UK; 4 J. Craig Venter Institute, Rockville, MD USA; 5 Noblis, Reston, VA USA; 6 Department of Medicine, Faculty of Medicine Siriraj Hospital, Mahidol University, Bangkok, Thailand

**Keywords:** *Klebsiella pneumoniae* isolates, Carbapenemase-producing *Enterobacteriaceae*, Antimicrobial resistance genes, Patient outcomes, Virulence genes, Phylogenetics

## Abstract

**Background:**

Infections caused by carbapenemase-producing *Enterobacteriaceae* (CPE) have continually grown as a global public health threat, with significant mortality rates observed across the world. We examined the clinical data from patients with CPE infections and their outcomes, concentrating on *Klebsiella pneumoniae* isolates. We analysed the clinical information, performed antimicrobial susceptibility testing, and conducted molecular epidemiological and genomic analyses on the isolates to identify patterns in the data.

**Methods:**

The clinical characteristics of 33 hospitalised patients with confirmed CPE, including patient-related factors associated with the development of CPE infections, were examined. Patients were divided according to whether they were “colonised” or “infected” with CPE and by the timing and frequency of their rectal swab collections, from which 45 swabs were randomly selected for analysis. CPE isolates were purified, and antimicrobial susceptibility tests performed. Whole genome sequences of these isolates were determined and analysed to compute bacterial multilocus sequence types and plasmid replicon types, infer phylogenetic relationships, and identify antimicrobial resistance and virulence genes.

**Results:**

Altogether, 88.9% (40/45) of the CPE isolates were *K. pneumoniae*. The most abundant carbapenemase gene family in the *K. pneumoniae* isolates (33/39) was *bla*_OXA-232_, with *bla*_NDM-1_ additionally identified in 19 of them. All CPE isolates carrying either *bla*_OXA-232_ or *bla*_NDM-1_ were resistant to meropenem, but only 40 from 45 were susceptible to colistin. Among the CPE-infected patients (*n* = 18) and CPE-colonised patients who developed CPE infections during the study (*n* = 3), all but one received standard colistin-based combination therapy. Phylogenetic analysis revealed the polyclonal spread of carbapenemase-producing *K. pneumoniae* (CPKP) within the patient population, with the following two major subclades identified: ST16 (*n* = 15) and ST231 (*n* = 14). CPKP-ST231 had the highest virulence score of 4 and was associated with primary bacteraemia. The siderophores yersiniabactin and aerobactin, considered to be important virulence factors, were only identified in the CPKP-ST231 genomes.

**Conclusions:**

This study has revealed the genomic features of colonising CPE isolates, focusing on antimicrobial resistance and virulence determinants. This type of multi-layered analysis can be further exploited in Thailand and elsewhere to modify the regimes used for empirical antibiotic treatment and improve the management strategies for CPE infections in hospitalised patients.

**Supplementary Information:**

The online version contains supplementary material available at 10.1186/s12879-021-05790-9.

## Background

Infections caused by carbapenemase-producing *Enterobacteriaceae* (CPE), which have increased worldwide in number to become a significant clinical problem over the last decade, are associated with high morbidity and mortality [1]. An early longitudinal study from Asia (2000–2012) revealed the prevalence of CPE was extremely low with average rates between 0.6–0.9% [2]. However, a survey from the National Antimicrobial Resistance Surveillance, Thailand from 2008 to 2016 revealed that carbapenemase-producing *Klebsiella pneumoniae* (CPKP) had increased in prevalence from 0.4% in 2008 to 5.4% in 2016 [3]. A recent retrospective cohort study from a 1200-bed university hospital in Bangkok reported on an increased incidence of CPE from 3.37 per 100,000 patient-days in 2011 to 32.49 per 100,000 patient-days between 2011 and 2016 [4]. The resistance mechanism for CPE is attributed to the following Ambler molecular classes of carbapenem-hydrolysing beta-lactamases: class A (KPC), class B (IMP, NDM, VIM), and class D (OXA-48) [5]. There is insufficient data from Thailand on the distribution of beta-lactamase (*bla*) genes [6, 7]. However, a recent study from a university hospital in Bangkok revealed that *bla*_NDM_ was the most common such gene followed by *bla*_OXA-48-like_ alleles (e.g., *bla*_OXA-48_, *bla*_OXA-181_, and *bla*_OXA-232_) [7]. Among all *Enterobacteriaceae*, CPKP is commonly associated with numerous antimicrobial resistance (AMR) genes and virulence determinants [8]. CPKP with its plasmid-encoded carbapenemases (e.g., *bla*_NDM_ and *bla*_KPC_) and its multiclass antibiotic resistance is associated with hospital-acquired infections and treatment challenges [9, 10]. Hypervirulent *K. pneumoniae* (hvKP) can also cause invasive diseases such as liver abscesses and metastatic infections [11]. In addition to having the K1 capsular serotype, hvKP encodes other virulence determinants (e.g., yersiniabactin, aerobactin, and salmochelin siderophores), and *rmp*A1/*rmp*A2 genes, which upregulate capsule expression and are associated with more invasive infections [8, 12, 13].

Although genetic diversity in carbapenemases has previously been reported in Thailand [7, 14], the epidemiology and characteristics of CPKP and its virulence determinants are not as well understood. Information is also lacking on the role played by hypervirulent strains of CPKP in hospital-acquired infections and how specific virulence determinants are associated with AMR profiles, disease severity and outcomes among hospitalised patients. The aim of the present study was to investigate the molecular epidemiological features of CPKP and its association with the clinical presentations of CPKP-infected patients. We also aimed to identify virulence determinants in the CPKP strains isolated from patients in this study.

## Methods

### Patient population and specimen collection

Eligible patients included all hospitalised patients aged ≥18 years who had CPE recovered from clinical specimens submitted to the microbiology laboratory. The participants were classified as CPE-colonised patients or CPE-infected patients at enrolment. CPE-colonised patients were defined according to whether or not CPE carriage was found, but its presence was not associated with any symptoms or clinical disease. CPE-infected patients were defined according to whether or not CPE isolates were found at sites of infection diagnosed as CPE infections by clinicians. Rectal swabs were collected from the patients within 48 h of enrolment and then once a week until the specimens were CPE-negative for 3 consecutive weeks (at the time of study there was no routine CPE screening procedure). One hundred and nineteen patients met the eligibility criteria, including 69 CPE-colonised patients and 50 CPE-infected patients at enrolment. Forty-five randomly selected and deidentified rectal swab samples from 33 patients, who were admitted for treatment between December 2015 and April 2017, were used for bacterial isolation and subsequent whole genome sequencing. Clinical information from each patient was collected, including information on patient demographics, clinical diagnosis, as well as their treatment during hospitalisation and outcome. The clinical outcomes of the CPE-infected patients at the end of treatment were classified as either ‘favourable response’ (absence or improvement of all clinical signs and symptoms of CPE infection) or ‘unfavourable response’ (worsening or persistence of clinical signs and/or symptoms of CPE infection, superinfection, or death).

### Bacterial isolates and antimicrobial susceptibility testing

Rectal swabs were inoculated onto MacConkey agar (BD, USA) supplemented with ceftriaxone (4 mg/L) and the plates incubated at 37 °C for 18 h. Bacterial identification was performed using Biotyper MALDI-TOF MS (Bruker Daltonics, Germany) according to the manufacturer’s protocol. Colonies identified as *Enterobacteriaceae* were tested for antimicrobial susceptibility using standard methods and following the guidelines for the disk-diffusion method [15]. Confirmation of suspected carbapenemase production in *Enterobacteriaceae*-positive specimens was performed using a modified carbapenem inactivation method [16]. Phenotypic screening for the presence of carbapenemases was performed using a double-disc synergy approach with phenylboronic acid or ethylenediaminetetraacetic acid with meropenem as previously described [17]. Colistin resistance was tested using the broth microdilution method with cation-adjusted Mueller–Hinton II broth [16]. Susceptibility to tigecycline was not tested at Siriraj Hospital during the study period.

### Genome sequencing and analysis

DNA was extracted using the Wizard® Genomic DNA Purification Kit (Promega, USA) and the multiplexed Illumina NexteraXT-generated libraries prepared from it were sequenced on the NextSeq® 500 platform (Illumina, USA) using a 2 × 150-bp paired-end kit with an average target coverage of 100-fold. Each read set was de novo assembled using SPAdes [18] and annotated with NCBI’s prokaryotic genome annotation pipeline (PGAP) [19] as described previously [20, 21]. For phylogenetic analysis, reads were mapped to the *K. pneumoniae* QS17–0029 (GCA_003073235.1) reference genome, which was previously identified in Thailand and known to carry *mcr*-1 [22], using Snippy v3.0 (https://github.com/tseemann/snippy). FastTree v2.1 [23] was used to generate an approximate maximum-likelihood phylogenetic tree. Metadata and phylogenetic trees were visualised using iTOL v4 [24]. To identify the bacterial sequence types, AMR genes, virulence loci and plasmid replicon types, the assembled contigs were analysed by multilocus sequence typing (MLST)1.8 [25], ResFinder3.1.0 [26], the Comprehensive Antibiotic Research Database [27], Kleborate3.0 (https://github.com/katholt/Kleborate), and PlasmidFinder2.0 [28], respectively. Virulence loci (yersiniabactin, *ybt*; colibactin, *clb*; salmochelin, *iro*; aerobactin, *iuc*; hypermucoidy, *rmp*A1, *rmp*A2; *Klebsiella* capsule K-locus, KL; and ICEKp-associated virulence loci in CPKP) were identified using Kleborate v3.0 (https://github.com/katholt/Kleborate). The virulence scores, which ranged from 0 to 5, were calculated as follows: 0 = no virulence loci; 1 = yersiniabactin only; 2 = yersiniabactin and colibactin, or colibactin only; 3 = aerobactin and/or salmochelin only (without yersiniabactin or colibactin); 4 = aerobactin and/or salmochelin with yersiniabactin (without colibactin); and 5 = yersiniabactin, colibactin and aerobactin and/or salmochelin.

## Results

### Clinical characteristics of the study participants

Thirty-three patients had their CPE isolates whole genome sequenced for this study. Of these, 15 patients were found to be colonised with CPE, while the others had a confirmed CPE infection at the time of enrolment. Nineteen of the 33 patients (57.6%) were female. The mean age of all the patients was 62.8 years (IQR, interquartile range: 47–81 years) (Table 1). Most CPE-colonised patients (*n* = 13/15) had experienced prolonged hospitalisation before CPE was detected in their rectal swabs (median stay: 21 days, IQR 0–34 days), and 39% of them were admitted to the intensive care unit. Thirty-two patients had underlying conditions such as diabetes mellitus or chronic kidney disease and had received antibiotic treatment within the month prior to enrolment, of which two thirds (*n* = 23) received carbapenems (Supplementary Table 1). Among the 15 CPE-colonised patients, three (16.7%) had CPE colonisation detected prior to developing CPE infection during their hospital stay (median 16 days, IQR; 3–31.75 days) (Supplementary Table 1). We still included these three patients in the CPE-colonised group. Among the CPE-infected patients, ventilator-associated pneumonia (VAP) was the most common consequence of CPE infection, followed by urinary tract infection and primary bacteraemia (Supplementary Table 1). Only one CPE-infected patient received colistin monotherapy; all 17 of the other patients received colistin-based combination therapy, with the median duration of antibiotic treatment lasting 11 days (IQR: 7–14 days) (Supplementary Table 1). Colistin–fosfomycin was the most common antimicrobial combination regimen (45%, *n* = 9/20) followed by colistin–piperacillin/tazobactam (15%, *n* = 3/20) (Table 1). Colistin–fosfomycin was the first treatment option for patients with carbapenem-resistant infections because this combination has been shown to afford higher microbiological eradication rates than colistin monotherapy in Siriraj Hospital [29]. Regarding the local antibiogram, because CPE was more susceptible to piperacillin–tazobactam than to imipenem and meropenem, the second most common combination regime was colistin–piperacillin/tazobactam. Unfavourable clinical outcomes were observed in 52.4% of all the CPE-infected patients (*n* = 21), including three who were initially colonised with CPE but later developed CPE infections, and seven of these patients experienced superinfections with different bacterial species at the end of their antibiotic regimes (Table 1). There was no statistically significant mortality observed between the CPE-infected patients (47.6%, *n* = 10/21) vs. the CPE colonised ones (33.3%, *n* = 4/12; chi-square test; *p* = 0.43).

**Table 1:** Demographic data of 33 patients reported in the present study and the results of antimicrobial susceptibility testing for the associated 45 carbapenemase-producing *Enterobacteriaceae* (CPE) isolates

		Demographic data of patients				Characteristic of isolated bacteria				Antimicrobial susceptibility testing results			
Patient No.	Age group	Ward	Treatment	Outcome	Hospital mortality	Strain ST	Taxonomy	Collection date	Type	MER	AMK	COL	FOS
1	E	IM	COL+FOS	Nonresponse	Death	KPCRETH01 (14)	*K. pneumoniae*	15/12/2015	Co	R	R	S	S
						KPCRETH05 (14)	*K. pneumoniae*	22/12/2015	Co	R	R	S	S
2	E	ICU	COL+PIP	Nonresponse	Death	KPCRETH06 -	*K. quasipneumoniae*	01/04/2016	Co	R	R	S	R
						ECCRETH01 (14)	*K. pneumoniae*	28/01/2016	Co	R	R	S	R
3	E	IM	No treatment	Not applicable	Alive	ECCRETH02 (410)	*E. coli*	21/12/2015	Co	R	R	S	S
4	E	IM	No treatment	Not applicable	Alive	KPCRETH07 (16)	*K. pneumoniae*	08/01/2016	Co	R	R	S	S
5	E	IM	No treatment	Not applicable	Alive	KPCRETH02 (16)	*K. pneumoniae*	01/09/2016	Co	R	I	S	R
6	E	IM	No treatment	Not applicable	Death	KPCRETH08 (70)	*K. pneumoniae*	17/02/2016	Co	R	R	S	I
						KPCRETH09 (16)	*K. pneumoniae*	23/02/2016	Co	R	R	S	I
7	E	IM	No treatment	Not applicable	Death	KPCRETH10 (37)	*K. pneumoniae*	08/02/2016	Co	R	R	S	R
8	E	IM	No treatment	Not applicable	Death	KPCRETH11 (16)	*K. pneumoniae*	17/02/2016	Co	R	S	S	R
9	OA	ICU	COL+TIG	Superinfection	Alive	KPCRETH12 (16)	*K. pneumoniae*	08/04/2016	Co	R	R	S	R
10	OA	IM	No treatment	Not applicable	Alive	KPCRETH13 (16)	*K. pneumoniae*	26/04/2016	Co	R	I	S	S
11	YA	ICU	No treatment	Not applicable	Alive	KPCRETH14 (231)	*K. pneumoniae*	11/05/2016	Co	R	R	S	I
						KPCRETH15 (231)	*K. pneumoniae*	27/05/2016	Co	R	R	S	I
12	E	ICU	No treatment	Not applicable	Alive	KPCRETH16 (231)	*K. pneumoniae*	17/05/2016	Co	R	R	S	R
13	E	ICU	No treatment	Not applicable	Death	ECCRETH03 (231)	*K. pneumoniae*	29/04/2016	Co	R	R	S	R
14	YA	IM	No treatment	Not applicable	Alive	ECCRETH04 (2003)	*E. coli*	18/08/2016	Co	R	R	S	S
						ECCRETH05 (2003)	*E. coli*	26/08/2016	Co	R	R	S	S
15	E	IM	No treatment	Not applicable	Alive	ECCRETH06 (410)	*E. coli*	28/11/2016	Co	R	S	S	R
						KPCRETH17 -	*E. hormaechei*	09/12/2016	Co	R	S	S	R
16	E	IM	COL+FOS	Response	Alive	KPCRETH03 (231)	*K. pneumoniae*	30/05/2016	Co	R	R	S	R
						KPCRETH04 (231)	*K. pneumoniae*	21/06/2016	Co	R	R	S	R
						KPCRETH26 (231)	*K. pneumoniae*	16/08/2016	Co	R	R	S	R
17	E	IM	COL+FOS	Response	Alive	KPCRETH18 (101)	*K. pneumoniae*	09/12/2015	In	R	R	S	I
						KPCRETH19 (101)	*K. pneumoniae*	05/01/2016	In	R	R	S	I
						KPCRETH20 (101)	*K. pneumoniae*	12/01/2016	In	R	R	S	I
18	E	ICU	COL+FOS	Nonresponse	Death	KPCRETH21 (231)	*K. pneumoniae*	24/03/2016	In	R	R	S	R
19	YA	ICU	COL+ERT	Response	Alive	KPCRETH22 (16)	*K. pneumoniae*	07/04/2016	In	R	S	S	I
						KPCRETH23 (16)	*K. pneumoniae*	11/05/2016	In	R	S	S	I
						KPCRETH24 (16)	*K. pneumoniae*	18/05/2016	In	R	S	S	I
20	OA	IM	COL	Response	Death	KPCRETH25 (16)	*K. pneumoniae*	27/05/2016	In	R	I	S	I
21	OA	IM	COL+MER	Response	Alive	KPCRETH27 (231)	*K. pneumoniae*	10/06/2016	In	R	R	S	R
22	YA	ICU	COL+PIP	Nonresponse	Death	KPCRETH28 (16)	*K. pneumoniae*	16/06/2016	In	R	I	S	R
23	YA	IM	COL+ FOS	Response	Alive	KPCRETH29 (14)	*K. pneumoniae*	22/06/2016	In	R	R	S	R
24	OA	IM	COL+PIP	Superinfection	Death	KPCRETH30 (14)	*K. pneumoniae*	17/05/2016	In	R	S	S	I
25	E	ICU	COL+ FOS	Superinfection	Death	KPCRETH31 (231)	*K. pneumoniae*	24/06/2016	In	R	R	S	I
26	E	IM	COL+ FOS	Response	Alive	KPCRETH32 (231)	*K. pneumoniae*	11/08/2016	In	R	R	S	R
27	OA	IM	COL+FOS	Superinfection	Death	KPCRETH33 (231)	*K. pneumoniae*	10/10/2016	In	R	S	S	R
28	OA	IM	COL+ FOS	Response	Alive	ECCRETH07 (231)	*K. pneumoniae*	15/12/2016	In	R	S	S	S
29	OA	IM	COL+FOS + GEN	Superinfection	Death	KPCTRPRTH01 (16)	*K. pneumoniae*	05/12/2016	In	R	R	R	R
30	OA	ICU	COL+ LEV	Superinfection	Death	KPCTRPRTH02 (231)	*K. pneumoniae*	03/01/2017	In	R	R	R	R
31	E	ICU	COL+FOS + GEN	Response	Alive	KPCTRPRTH03 (231)	*K. pneumoniae*	23/01/2017	In	R	R	R	R
32	OA	ICU	COL+MER	Superinfection	Death	KPCTRPRTH04 (16)	*K. pneumoniae*	17/04/2017	In	R	R	R	R
33	OA	ICU	COL+MER + LEV	Response	Alive	KPCTRPRTH05 (16)	*K. pneumoniae*	20/04/2017	In	R	R	R	R

*YA* Young adult (18–39 years), *OA* Older adult (40–64 years), *E* Elderly (65+ years), *IM* Internal medicine ward, *yrs*. Years, *ICU* Intensive care unit, *MER* Meropenem, *AMK* Amikacin, *COL* Colistin, *ERT* Ertapenem, *FOS* Fosfomycin, *PIP* Piperacillin–tazobactam, *TIG* Tigecycline, *GEN* Gentamicin, *LEV* Levofloxacin, *NR* Nonresponse: worsening or persistence of clinical signs and/or symptoms of CPE infection, *R* Response: absence or improvement of all clinical signs and symptoms of CPE infection, *SI* Superinfection: second infection superimposed on an earlier one by different bacteria, *Co* Colonisation, *In* Infection, *S* Susceptible, *I* Intermediate, *R* Resistant

### Antimicrobial susceptibility patterns detected in the CPE isolates

We isolated 39 *K. pneumoniae*, four *Escherichia coli*, and one isolate each of *Enterobacter hormaechei subsp. steigerwaltii* and *K. quasipneumoniae subsp. similipneumoniae* from the 33 patients in our study (Table 1). All 45 isolates displayed meropenem resistance, only 20% of them (*n* = 9) were susceptible to amikacin, and 17.8% (*n* = 8) were susceptible to fosfomycin (Table 1). All of the CPE isolates were resistant to ciprofloxacin, cefoxitin, ceftriaxone, ceftazidime, piperacillin–tazobactam, ertapenem and imipenem (Table 1). Only five of the isolates, all *K. pneumoniae*, showed resistance to colistin with MIC values ranging between 32 and 64 mg/L (Table 1). No significant differences between the antimicrobial susceptibility patterns of isolates from CPE-colonised patients and those from CPE-infected patients were observed, indicating that colonising and disease-causing strains show very similar AMR profiles, although this finding may also be attributed to the relatively small sample size available.

### High diversity in AMR genes and plasmids in the CPE isolates

**Fig. 1:**
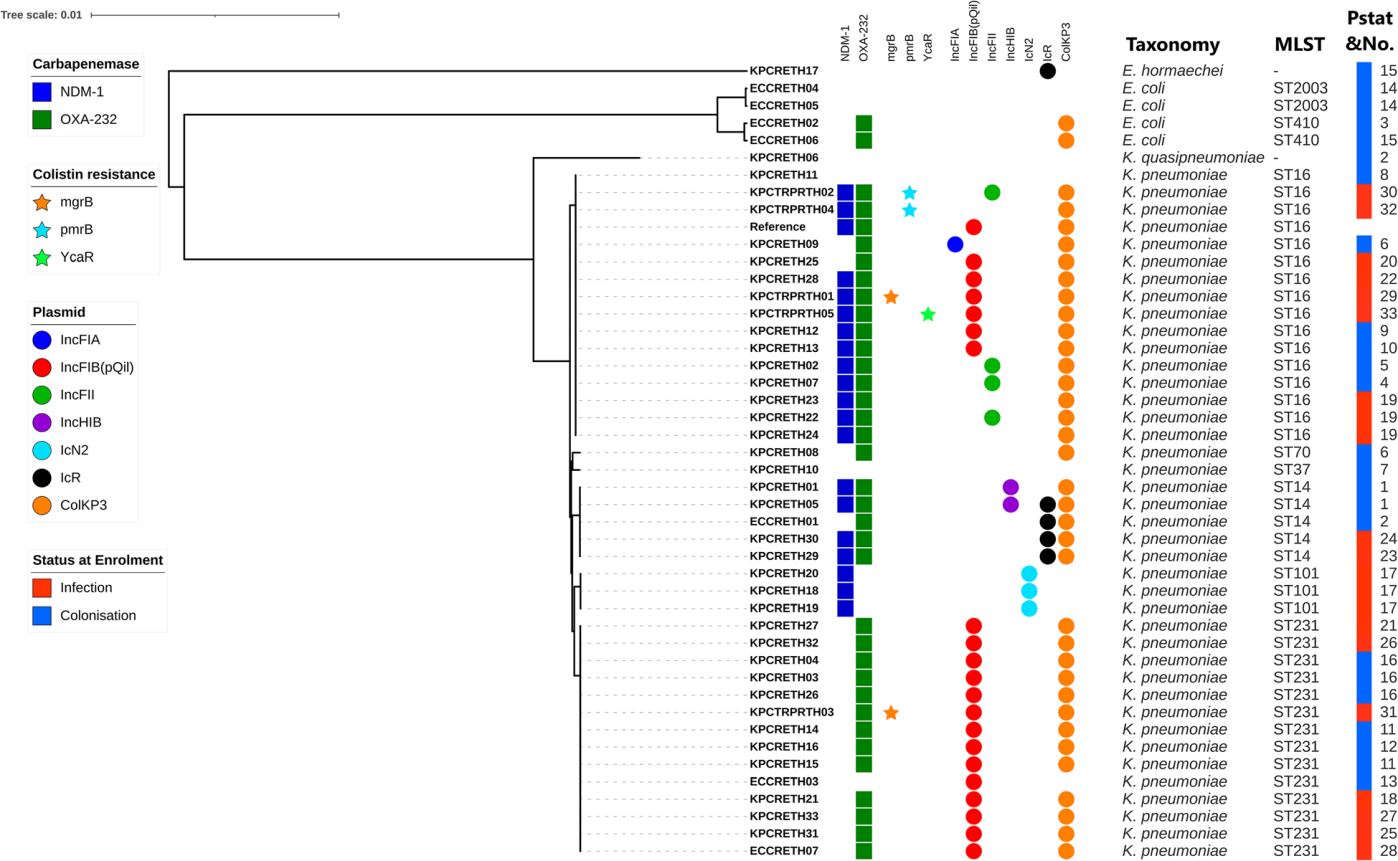
A phylogenetic tree showing the relationship between all 45 carbapenemase-producing *Enterobacteriaceae* (CPE) genomes studied. The panel on the right shows the number of properties of the genomes—namely (from left to right), the presence (coloured symbol) and absence (no symbol) of the selected antimicrobial resistance genes (beta-lactamase genes: *bla*_NDM-1_, *bla*_OXA-232_; colistin-resistant mutations present in: *mgr*B, *pmr*B, *Yca*R), plasmid replicons (circles), bacterial species, sequence type based on MLST profiles, patient’s status at enrolment and patient number (Pstat&No). The phylogenetic tree was based on 123,598 core SNPs of 45 CPE isolates using a reference genome (*K. pneumoniae* QS17–0029; NCBI no.: GCA_003073235.1). Scale bar indicates the number of nucleotide substitutions per site

Our genomic analysis showed that *bla*_OXA-232_ was the most dominant carbapenemase gene family and was found in 34 of 39 *K. pneumoniae* and two of the four *E. coli* isolates we sequenced (Supplementary Table 2). The two most common sequence types (STs) identified in *K. pneumoniae* were ST16 (*n* = 15) and ST231 (*n* = 14), from which 12 ST16 isolates carried *bla*_OXA-232_ and *bla*_NDM-1_, whereas almost all of the ST231 (*n* = 13) isolates carried only *bla*_*OXA-232*_ (Fig. 1, Supplementary Table 2). In addition, all of the CPE isolates carrying β-lactamase genes also carried genes encoding other AMR genes, including aminoglycosides (*aac*(6)-I, *aph* [3]), fluoroquinolones (*qnr*B, *qnr*S), and fosfomycins (*fosA6*, *Uhp*T) (Supplementary Table 2). None of the five colistin-resistant isolates harboured *mcr*-genes, although they were highly resistant to colistin (Table 1), We found a *pmr*B (D150Y) mutation in KPCTRPRTH02 and KPCTRPRTH04, *mgr*B disruptions were observed in KPCTRPRTH03 (W20*) and KPCTRPRTH01 (Q30*). In isolate KPCTRPRTH05, a S60L mutation in Y*ca*R was also identified (Brinkac et al., manuscript in preparation).

We identified the following range of incompatibility (Inc) plasmid groups in the CPE isolates: FIA, FIB (pQil), FII, HI2B, N2 and R (Fig. 1). We were particularly interested in the presence of IncFIB and the small-sized Col plasmid group in our CPE dataset because these two plasmid groups are reported to be most commonly found in clinical samples and are associated with the spread of AMR genes [30]. Interestingly, in our dataset, all cases where ST231-CPKP was present (*n* = 14) and nearly half of those with ST16-CPKP (*n* = 7) contained an IncFIB (pQil)-like plasmid (Fig. 1). Additionally, genomic analysis indicated that all *bla*_OXA-232_-containing CPE isolates were predicted to contain ColKp3 plasmid replicons (Fig. 1).

### *K. pneumoniae* isolates carry genes associated with hypervirulence

**Fig. 2:**
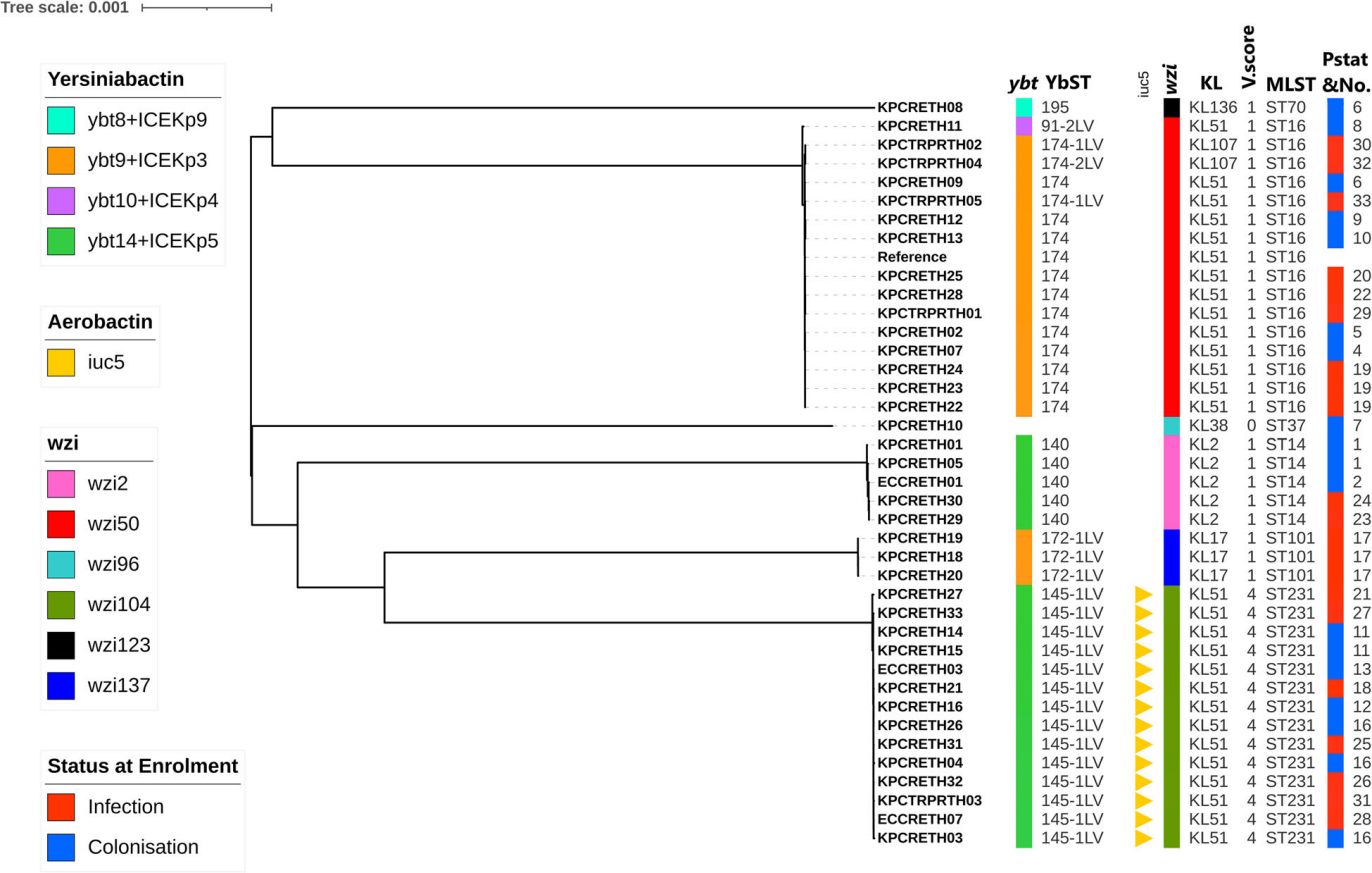
A phylogenetic tree showing the relationship between 39 carbapenemase-producing *Klebsiella pneumoniae* (CPKP) isolates. The panel on the right shows a number of properties of the genomes—namely (from left to right), the presence or absence of virulence determinants such as *ybt* and ICE*Kp*, *ybt* sequence type (YbST), *iuc*, *wzi* allele, KL and virulence score (V.score); sequence type based on MLST profiles; patient’s status at enrolment and patient number (Pstat&No). The phylogenetic tree is based on 70,571 core SNPs of the 39 CPKP isolates using a reference genome (*K. pneumoniae* QS17–0029; GCA_003073235.1). Scale bar indicates the number of nucleotide substitutions per site

We searched the *K. pneumoniae* genomes for the virulence genes previously found in hvKP strains, including those encoding siderophores for the biosynthesis and uptake of iron (*ybt*, *iuc* and *iro*) and genes for the regulator of mucoid phenotype (*rmp*A1/*rmp*A2) [13]. The *ybt* locus, encoding the siderophore yersiniabactin, was present in 38/39 of the CPKP genomes. The most common allele, *ybt*14 (located on ICE*Kp*5), was identified in 19 isolates, while the second most common allele, *ybt*9 (located on ICE*Kp3*), was identified in 17 isolates, and the rest two isolates had *ybt*8 (located on ICEKp9) and *ybt*10 (located on ICEKp4), respectively (Fig. 2). Notably, *iuc*5, encoding the siderophore aerobactin, was only detected in ST231 (*n* = 14). We detected six distinct K locus (KL) types among 39 CPKP isolates, the most frequent ones being KL51 (*n* = 28), KL2 (*n* = 5), and KL17 (*n* = 3) (Fig. 2). Virulence plasmid-associated loci such as *iro*, encoding the siderophore salmochelin, colibactin and *rmp*A1 and *rmp*A2 were not present in the investigated CPKP genomes. We also found that *wzi*50 was more common in the ST16 isolates, whereas *wzi*104 was only found in the ST231 isolates, and capsular antigen KL51 was found in ST16 and ST231 isolates alike.

### Associations between the patients’ clinical data and the CPE isolates

We identified nine patients who had more than one CPE isolate isolated throughout their hospital stay (Table 1). Six of them, despite receiving appropriate treatment, had > 2 follow-up isolates that were the same bacterial species with the same sequence type and similar antibiogram pattern (Table 1). Genomic analysis also confirmed that the bacterial isolates from the same patient were identical with only 0–1 single-nucleotide polymorphism (SNP) difference.

We noted that the bla_OXA-232_ carbapenemase-encoding ColKP3 plasmid was present in different strains of *K. pneumoniae* as well as in *E. coli* **(**Fig. 1**)**, indicating the possibility of horizontal interspecies spread of this plasmid and possibly resulting in a polyclonal outbreak within our hospital. Although ST231 and ST16 were the two main clones associated with invasive disease and poor outcomes in our study, we did not identify any particular STs that were found only in CPE-colonised patients or only in CPE-infected patients.

## Discussion

To the best of our knowledge, this is the first study to document detailed molecular bacterial isolate information on carbapenem resistance, plasmid replicons, and virulence determinants in relation to the clinical characterisation of hospitalised patients in Thailand. Of the 25 CPE-patients with follow-up rectal swab cultures, the mean time to culture negativity was 37.7 days in our study. This finding is consistent with previous reports that 54% of CPE rectal carriers remain CPE carriers for 30 to 60 days after their initial screening, 28% remain as such after 6 months to 1 year, and 14% remain as such after 1 year [31, 32]. In our study, approximately 16.7% of the asymptomatic rectal carriers developed a clinical infection with a median duration of 20 days. The incidence of CPE infections in the CPE-colonised patients in our study was as high as that seen previously [33, 34], and there are several possible explanations for this. One explanation is that we began to observe patients who already had CPE colonisation at sites other than the gut, which might be a risk factor for them developing clinical infections [35]. Another explanation is that most of the patients had multiple comorbidities (e.g., diabetes mellitus and renal diseases) resulting in prolonged hospitalisation, possibly predisposing them to CPE colonisation and subsequent CPE infection.

Antimicrobial susceptibility testing in our study confirmed resistance to piperacillin/tazobactam, ciprofloxacin and meropenem in all the CPE isolates. Moreover, only 17.8% of the CPE strains isolated from the patients were susceptible to fosfomycin. Colistin is presumably the most active agent against up to 89% of the CPE isolates from our study. The evidence from a cohort study [26] and systematic review [13] on antibiotic therapy in CPE infections revealed that combination therapy is probably more effective than monotherapy. Therefore, the antibiotic therapy recommendation for CPE infections at Siriraj Hospital is combination therapy, with colistin acting as the backbone of the regimen.

In our study, the mortality rate was 47.6% for CPE-infected patients and 33.3% for patients colonised with CPE. The difference was not statistically significant. However, attributable mortality is difficult to assess because both groups already had high overall mortality and the sample size was small.

Although KPC-producing *Enterobacteriaceae* are reported to have spread rapidly over the last decade, their prevalence in Thailand remains very low [6, 7]. Notably, the CPE prevalence was 1.4%, and *bla*_KPC-13_ and *bla*_IMP-14_ were the only carbapenemase genes detected among the CPE isolates identified at Siriraj Hospital during 2009 to 2011 [6]. However, the incidence of CPE bacteraemia has significantly increased from < 1% in 2011 to 3.8% in 2017 [6, 36]. The main CPE identified herein was CPKP, which carried one carbapenemase gene (*bla*_NDM-1_ or *bla*_OXA-232_) and at least one other *bla* gene. *bla*_OXA-48_-like genes were the most common carbapenemase genes, with *bla*_OXA-232_ detected in 78% of the isolates. We also found that 46% of the *bla*_OXA-232_ isolates also carried *bla*_NDM-1_, a finding consistent with that reported previously in Thailand [7]. This highlights that isolates with *bla*_OXA-48_-like genes continue to be a problem in Thailand.

The previously reported cases of *bla*_OXA-232_ –harbouring *K. pneumoniae* were mainly serotypes ST14 and ST231 [37–39], while ST16 and ST231 were the dominant epidemic serotypes in our study. Thus, ST231 may be a high-risk, carbapenem-producing *K. pneumoniae* clone actively disseminating across Southeast Asia, with related outbreaks being reported in Switzerland [40]. We found that all 36 *bla*_OXA-232_-harbouring isolates were present on a small ColKP3 plasmid in our dataset of *E. coli* and *K. pneumoniae* genomes, a finding concordant with that from a previous report [37]. Interestingly, IncFIB(pQil) plasmids were identified in all ST-231 *K. pneumoniae* isolates in our study, and both ColKP3 and IncFIB(pQil) are known to carry *bla*_OXA-232_ and *bla*_TEM-1_ [41, 42]. These findings confirm that both plasmids, IncFIB(pQil) and ColKP3, are often found in clinical isolates and contain multiple AMR genes, as has been previously reported [30].

Among our CPKP isolates, we found two virulence loci that have been previously associated with invasive diseases: *ybt* and *iuc*, encoding the siderophores yersiniabactin and aerobactin, respectively [8, 12, 43]. *Ybt* was found in almost all of our CPKP isolates (97.4%), and all *ybt* loci detected in the CPKP genomes were associated with an ICE*Kp* structure located in a chromosomal region [12]. ICE*Kp*, an integrative conjugative element, is self-transmissible and occasionally contains virulence factors such as *ybt* and *iro* [12]. Thus, ICE*Kp* is considered to be an important mediator of pathogenicity in *K. pneumoniae* [12]. VAP was the most common CPKP infection, and since all isolates from patients with VAP had *ybt*, these findings raise the interesting possibility that the yersiniabactin siderophore can promote respiratory tract infection as previous studies [44, 45]. This is the first identification of *iuc*5 in ST231-CPKP isolates in Thailand and Southeast Asia; otherwise, *iuc*5 has only been found in ST231-CPKP from India [46]. Some KL types (e.g., KL1, KL2, KL5 and KL57) are considered to be hypervirulent variants of *K. pneumoniae* and are associated with invasive diseases [47]. Our results show that there were at least seven distinct *Klebsiella* capsule genes/loci present among the 39 isolates, from which KL51 was the most common. However, only 5 out of 39 of our isolates were KL2 types and all of them belonged to ST14, a non-hypervirulent clone usually encountered in hospital-acquired infections [48]. Our results also revealed that *wzi* alleles were associated with the expected MLSTs more than with KL types.

Of note, when we integrated the clinical information with the bacterial genomic data, we identified ST231-CPKP as the most common pathogen in CPE-infected patients, 6 out of 11 of which had invasive diseases such as primary bacteraemia and pneumonia. According to our analysis of virulence determinants, ST231-CPKP had the highest virulence score (Fig. 2) and contained *iuc*5. The *iuc* locus has been increasingly detected in hvKP over the last couple of years and is considered to be one of the most prominent features of invasive isolates [46, 49]. Second, four out of five of the colistin-resistant CPKP isolates belonged to ST16. ST16-CPKP with colistin resistance was found in CPE-infected patients presenting with VAP and three of these patients died while in hospital. Therefore, ST16-CPKP is considered to be one of the more clinically significant clones in our study, as was also reported elsewhere [50]. Third, the core SNP-based phylogenetic tree suggests the possibility of a polyclonal outbreak of CPKP, predominantly involving ST231 and ST16 CPKP in Siriraj Hospital between 2015 and 2017. We identified two major subclades of CPKP: ST231 (*n* = 15) and ST16 (*n* = 14). Lastly, ST101 and ST14 were identified among the CPE-infected patients, something previously reported in South and Southeast Asia [46, 51].

Several limitations in our study require mentioning. First, the strains examined were all isolated from rectal swabs, and CPEs that caused infections were not characterised. However, we collected the rectal swabs while the patients were infected with CPEs and the antibiograms of CPE isolates at the sites of infection were similar to the antibiograms of CPE isolates from the rectal swabs. Next, it was not possible to identify the risk factors potentially associated with poor outcomes because of the small sample size that was available in this study. We have probably overestimated the true prevalence of CPE colonisation because there was a lack of routine screening for CPE in patients on admission during the study period. Nevertheless, our results suggest some clinical correlations between the clinical outcomes of patients with CPE infections and the genomic analysis of the organisms responsible, and also provide essential epidemiological data that could be used to guide empirical treatment and infection control strategies for CPE patients.

## Conclusion

Our study represents the first report of a genomic epidemiological investigation on CPE among hospitalised patients in Thailand. By analysing resistance and virulence genes in combination with clinical patient information and bacterial genetic diversity, our approach provides important information that can be used to promptly track the emergence and spread of clinically significant isolates, suggest empirical antibiotics, assess mechanisms of drug resistance, and guide infection control strategies for CPE. Future larger-scale studies are needed to determine the true prevalence of CPE and to identify the risk factors for CPE acquisition and their impact on treatment outcomes in Thailand.

## Supplementary Information

**Additional file 1: Table S1.** Detailed clinical information of 33 carbapenemase-producing *Enterobacteriaceae* (CPE) patients described in the study. **Table S2.** Antimicrobial resistance determinants for 45 carbapenemase-producing *Enterobacteriaceae* isolates identified from whole genome sequencing analysis.

## Data Availability

Raw sequencing reads and assemblies are deposited in GenBank and the Sequence Read Archive under project accession: PRJNA389557. The biosample ID of each strain is shown in Supplementary Table 2.
