# Supplementary material for: Genomic and clinical characterisation of multidrug-resistant carbapenemase-producing ST231 and ST16 Klebsiella pneumoniae isolates colonising patients at Siriraj hospital, Bangkok, Thailand from 2015 to 2017
Source: BMC Infect Dis. 2021 Feb 4;21:142. doi: 10.1186/s12879-021-05790-9 (PMC7859894; doi:10.1186/s12879-021-05790-9)
Supplement: Supplementary file 1 — Additional file 1: Table S1. Detailed clinical information of 33 carbapenemase-producing Enterobacteriaceae (CPE) patients described in the study. Table S2. Antimicrobial resistance determinants for 45 carbapenemase-producing Enterobacteriaceae isolates identified from whole genome sequencing analysis. [file 12879_2021_5790_MOESM1_ESM.docx]

**Supplementary Table 1.** Detailed clinical information of 33 carbapenemase-producing *Enterobacteriaceae* (CPE) patients described in the study

| **Patient no.** | **Comorbid diseases** | **LOS (days)** | **CPE status** | **Prior ATB^1^** | **Time before colonisation (days)** | **Duration of colonisation (days)** | **Develop CPE infection** | **Duration between colonisation and infection (days)** | **Site of infection** | **Source** | **Treatment** | **Duration of treatment (days)** | **Strain** |
| --- | --- | --- | --- | --- | --- | --- | --- | --- | --- | --- | --- | --- | --- |
| 1 | Hypertension, Chronic kidney disease | 102 | Colonisation | LEV | 53 | 50 | Yes | 20 | Urinary tract infection | Urine | COL+FOS | 7 | KPCRETH01 KPCRETH05 |
| 2 | DM, Congestive heart failure, Stroke | 140 | Colonisation | IMI | 90 | 50 | Yes | 37 | Central line–associated bloodstream infection | Blood | COL+PIP | 13 | KPCRETH06 ECCRETH01 |
| 3 | Neuromyelitis optica | 44 | Colonisation | PIP+ MER | 34 | 13 | No | Not applicable | Not applicable | Urine | Not applicable | Not applicable | ECCRETH02 |
| 4 | Colon cancer | 19 | Colonisation | VAN+MER | 93 | NA | No | Not applicable | Not applicable | Urine | Not applicable | Not applicable | KPCRETH07 |
| 5 | DM, Ovarian cancer | 28 | Colonisation | PIP+ MER+MET | 18 | 10 | No | Not applicable | Not applicable | Abdominal fluid from percutaneous drainage | Not applicable | Not applicable | KPCRETH02 |
| 6 | DM, Congestive heart failure, Chronic kidney disease | 90 | Colonisation | PIP+ MER+FOS | 17 | 75 | No | Not applicable | Not applicable | Sputum | Not applicable | Not applicable | KPCRETH08 KPCRETH09 |
| 7 | Liver cirrhosis | 17 | Colonisation | No | 17 | 4 | No | Not applicable | Not applicable | Urine | Not applicable | Not applicable | KPCRETH10 |
| 8 | Chronic kidney disease | 49 | Colonisation | VAN+LEV+COL | 25 | 25 | No | Not applicable | Not applicable | Urine | Not applicable | Not applicable | KPCRETH11 |
| 9 | DM | 95 | Colonisation | PIP+ COL | 5 | 91 | Yes | 20 | Ventilator-associated pneumonia | Sputum | COL+TIG | 13 | KPCRETH12 |
| 10 | Hepatocellular carcinoma, HIV | 22 | Colonisation | LEV+MER | 7 | NA | No | Not applicable | Not applicable | Sputum | Not applicable | Not applicable | KPCRETH13 |
| 11 | SLE | 49 | Colonisation | VAN+MER | 11 | 38 | No | Not applicable | Not applicable | Sputum | Not applicable | Not applicable | KPCRETH14 KPCRETH15 |
| 12 | Chronic kidney disease | 77 | Colonisation | VAN+CEZ | 21 | 38 | No | Not applicable | Not applicable | Urine | Not applicable | Not applicable | KPCRETH16 |
| 13 | Coronary artery disease, COPD | 78 | Colonisation | VAN+MER+COL | 39 | 25 | No | Not applicable | Not applicable | Urine | Not applicable | Not applicable | ECCRETH03 |
| 14 | SLE | 76 | Colonisation | VAN+MER | 21 | 56 | No | Not applicable | Not applicable | Urine | Not applicable | Not applicable | ECCRETH04 ECCRETH05 |
| 15 | DM, Chronic kidney disease | 63 | Colonisation | MER | 48 | NA | No | Not applicable | Not applicable | Urine | Not applicable | Not applicable | ECCRETH06 KPCRETH17 |
| 16 | Congestive heart failure, COPD | 120 | Infection | PIP | 0 | 101 | Yes | Not applicable | Ventilator-associated pneumonia | Sputum | COL+FOS | 10 | KPCRETH03 KPCRETH04 KPCRETH26 |
| 17 | DM, Chronic kidney disease | 113 | Infection | PIP | 0 | 70 | Yes | Not applicable | Urinary tract infection | Urine | COL+FOS | 21 | KPCRETH18 KPCRETH19 KPCRETH20 |
| 18 | Drug induced Immunodeficiency | 20 | Infection | BIA+ LEV | 0 | 19 | Yes | Not applicable | Hospital-acquired pneumonia | Sputum | COL+FOS | 17 | KPCRETH21 |
| 19 | Rheumatoid arthritis | 60 | Infection | MER+COL | 0 | 60 | Yes | Not applicable | Primary bacteraemia | Blood | COL+ERT | 14 | KPCRETH22 KPCRETH23 KPCRETH24 |
| 20 | DM, Chronic kidney disease, Congestive heart failure | 59 | Infection | MER | 0 | 23 | Yes | Not applicable | Urinary tract infection | Urine | COL | 5 | KPCRETH25 |
| 21 | Cholangiocarcinoma | 36 | Infection | IMI+ COL | 0 | 21 | Yes | Not applicable | Primary bacteraemia | Blood | COL+MER | 11 | KPCRETH27 |
| 22 | Haematologic malignancy | 24 | Infection | VAN+MER | 0 | 7 | Yes | Not applicable | Primary bacteraemia | Blood | COL+PIP | 7 | KPCRETH28 |
| 23 | SLE | 210 | Infection | MER+LEV+COL | 0 | 28 | Yes | Not applicable | Ventilator-associated pneumonia | Sputum | COL+ FOS | 10 | KPCRETH29 |
| 24 | SLE | 47 | Infection | MER | 0 | 17 | Yes | Not applicable | Ventilator-associated pneumonia | Sputum | COL+PIP | 13 | KPCRETH30 |
| 25 | DM | 30 | Infection | VAN+PIP | 0 | 19 | Yes | Not applicable | Hospital-acquired pneumonia | Sputum | COL+ FOS | 17 | KPCRETH31 |
| 26 | No | 120 | Infection | PIP | 0 | 58 | Yes | Not applicable | Urinary tract infection | Urine | COL+ FOS | 6 | KPCRETH32 |
| 27 | SLE, Chronic kidney disease | 39 | Infection | MER+COL | 0 | 17 | Yes | Not applicable | Ventilator-associated pneumonia, Secondary bacteraemia | Sputum | COL+ FOS | 14 | KPCRETH33 |
| 28 | HIV | 19 | Infection | MER | 0 | 19 | Yes | Not applicable | Urinary tract infection | Urine | COL+ FOS | 14 | ECCRETH07 |
| 29 | DM, Haematologic malignancy | 29 | Infection | IMI | 0 | NA | Yes | Not applicable | Ventilator-associated pneumonia, Secondary bacteraemia | Sputum,  Blood | COL+FOS+ GEN | 15 | KPCTRPRTH01 |
| 30 | DM, Evans syndrome | 88 | Infection | VAN+MER+COL | 0 | NA | Yes | Not applicable | Ventilator-associated pneumonia | Sputum | COL+ LEV | 7 | KPCTRPRTH02 |
| 31 | Stroke, COPD | 76 | Infection | MER+LEV | 0 | NA | Yes | Not applicable | Ventilator-associated pneumonia | Sputum | COL+FOS+ GEN | 6 | KPCTRPRTH03 |
| 32 | Dyslipidaemia | 47 | Infection | PIP | 0 | NA | Yes | Not applicable | Ventilator-associated pneumonia | Sputum | COL+MER | 14 | KPCTRPRTH04 |
| 33 | SLE | 63 | Infection | MER | 0 | NA | Yes | Not applicable | Ventilator-associated pneumonia, Central line–associated bloodstream infection | Sputum,  Blood | COL+MER+ LEV | 14 | KPCTRPRTH05 |

ATB = antibiotic, CEZ = ceftazidime, BI = biapenem, ERT = ertapenem, IMI = imipenem, MER = meropenem, AMK = amikacin, COL = colistin, FOS = Fosfomycin, PIP = piperacillin–tazobactam, TIG = tigecycline, GEN = gentamicin, LEV= levofloxacin, VAN = vancomycin. NA = not available, COPD: chronic obstructive pulmonary disease, DM: diabetics mellitus, HIV: human immunodeficiency virus, SLE: Systemic lupus erythematosus.

**Supplementary Table 2**. Antimicrobial resistance determinants for 45 carbapenemase-producing *Enterobacteriaceae* isolates identified from whole genome sequencing analysis

| Strain | Taxonomy | Biosample ID | Beta-lactamase(bla) | Aminoglycoside | Fluoroquinolone | Fosfomycin |
| --- | --- | --- | --- | --- | --- | --- |
| KPCRETH01 | *K. pneumoniae* | SAMN07203007 | *bla*_OXA-9_, *bla*_CTX-M-15_, *bla*_OXA-1_, *bla*_OXA-232_, *_bla_*_TEM-1A_, *bla*_NDM-1_, *bla*_SHV-28_ | *aad*A2, *aph*(3')-VI, *aad*A1, *arm*A | *qnr*B1, *par*C(S80I), *gyr*A(D105G) | *fos*A6, *Uhp*T |
| KPCRETH02 | *K. pneumoniae* | SAMN07450551 | *bla*_CTX-M-15_, *bla*_NDM-1_, *bla*_TEM-1A_, *bla*_OXA-232_, *bla*_SHV-1_ | *aad*A2 | *gyrA*(D95N) | *fos*A6, *Uhp*T |
| KPCRETH03 | *K. pneumoniae* | SAMN07450550 | *bla*_OXA-232_, *bla*_TEM-1B_, *bla*_SHV-1_ | *aac*(6')-Ib, *aad*A2, | *qnr*S1, *par*C(S80I) | *fos*A6, *Uhp*T |
| KPCRETH04 | *K. pneumoniae* | SAMN07450592 | *bla*_OXA-232_, *bla*_TEM-1B_, *bla*_SHV-1_ | *aac*(6')-Ib, *aad*A2, | *qnr*S1, *par*C(S80I) | *fos*A6, *Uhp*T |
| ECCRETH01 | *K. pneumoniae* | SAMN07450591 | *bla*_CTX-M-15_, *bla*_OXA-232_, *bla*_OXA-1_, *bla*_SHV-28_ | *aac*(6')-Ib-cr | *par*C(S80I), *gyr*A(D105G) | *fos*A6 |
| ECCRETH02 | *E. coli* | SAMN07450589 | *bla*_CTX-M-55_, *bla*_OXA-232_ | *aph*(3')-Ia, *aph*(6)-Id | *par*C(S80I), *gyrA*(S101L) | *mdt*G |
| ECCRETH03 | *K. pneumoniae* | SAMN07203032 | *bla*_CTX-M-15_, *bla*_TEM-1B_, *bla*_SHV-1_ | *aac*(6')-Ib, *aad*A2, *rmt*F | *qnr*S1, *par*C(S80I) | *fos*A6 |
| ECCRETH04 | *E. coli* | SAMN07450588 | *bla*_CTX-M-55_ | *aph*(3')-IIa, *aph*(3'')-Ib, *aph*(6)-Id | *par*C(S80I), *gyr*A(S101L) | *mdt*G |
| ECCRETH05 | *E. coli* | SAMN07450587 | *bla*_CTX-M-55_ | *aph*(3')-IIa, *aph*(3'')-Ib, *aph*(6)-Id | *par*C(S80I), *gyr*A(S101L) | *mdt*G |
| ECCRETH06 | *E. coli* | SAMN07450586 | *bla*_CTX-M-14_, *bla*_CTX-M-15_, *bla*_CMY-2_, *bla*_TEM-1B_, *bla*_OXA-232_, *bla*_OXA-1_ | *aad*A22, *aad*A5, *aac*(6')-Ib-cr | *par*C(S80I), *gyr*A(S101L) | *mdt*G |
| ECCRETH07 | *K. pneumoniae* | SAMN07450584 | *bla*_CTX-M-15_, *bla*_TEM-1B_, *bla*_OXA-232_, *bla*_SHV-1_ | *aad*A2, *rmt*F | *qnr*S1, *par*C(S80I) | *fos*A6 |
| KPCRETH05 | *K. pneumoniae* | SAMN07450583 | *bla*_CTX-M-15_, *bla*_TEM-1A_, *bla*_NDM-1_, *bla*_OXA-232_, *_bla_*_OXA-1_, *bla*_OXA-9_, *bla*_SHV-28_ | *aad*A1, *aad*A2, *arm*A, *aph*(3')-VI | *qnr*B1, *par*C(S80I), *gyr*A(D105G) | *Uhp*T |
| KPCRETH06 | *K. quasipneumoniae* | SAMN07450576 | *bla*_OXA-10_, *bla*_OKP-B-3_, *bla*_IMP-14_ | *aph*(3')-VIa, *ant*(2'')-Ia | *par*C(A85T) | *Uhp*T |
| KPCRETH07 | *K. pneumoniae* | SAMN07450575 | *bla*_CTX-M-15_, *bla*_TEM-1A_, *bla*_NDM-1_, *bla*_OXA-232_, *bla*_SHV-1_ | *aad*A2 | *gyr*A(D95N) | *fos*A6, *Uhp*T |
| KPCRETH08 | *K. pneumoniae* | SAMN07450574 | *bla*_CTX-M-15_, *bla*_SHV-32_, *bla*_TEM-1A_, *bla*_OXA-232_, *bla*_OXA-9_ | *aad*A1, *aph*(3'')-Ib, *aac*(6')-Ib, *aph*(6)-Id | *qnr*B1, *par*C(A85T) | *Uhp*T |
| KPCRETH09 | *K. pneumoniae* | SAMN07450573 | *bla*_CTX-M-15_, *bla*_TEM-1A_, *bla*_OXA-232_, *bla*_OXA-9_, *bla*_SHV-1_ | *aad*A1, *aad*A2, *aac*(6')-Ib | *gyr*A(D95N) | *fos*A6, *Uhp*T |
| KPCRETH10 | *K. pneumoniae* | SAMN07450572 | *bla*_DHA-1_, *bla*_TEM-1B_, *bla*_SHV-11_ | *aad*A2, *aph*(3')-Ia | *qnr*B4, *par*C(S80I) | *Uhp*T |
| KPCRETH11 | *K. pneumoniae* | SAMN07450571 | *bla*_CTX-M-15_, *bla*_TEM-1B_, *bla*_OXA-1_, *bla*_SHV-1_ | *aad*A2, *aac*(6')-Ib-cr | *gyr*A(D95N) | *fos*A6, *Uhp*T |
| KPCRETH12 | *K. pneumoniae* | SAMN07450570 | *bla*_CTX-M-15_, *bla*_TEM-1A_, *bla*_NDM-1_, *bla*_OXA-232_, *bla*_OXA-9_, *bla*_SHV-1_ | *aad*A1, *aad*A2, *aac*(6')-Ib-cr | *qnr*B6, *gyr*A(D95N) | *fos*A6, *Uhp*T |
| KPCRETH13 | *K. pneumoniae* | SAMN07450569 | *bla*_CTX-M-15_, *bla*_TEM-1A_, *bla*_NDM-1_, *bla*_OXA-232_, *bla*_OXA-9_, *bla*_SHV-1_ | *aad*A1, *aad*A2 | *qnr*B6, *gyr*A(D95N) | *fos*A6, *Uhp*T |
| KPCRETH14 | *K. pneumoniae* | SAMN07450568 | *bla*_CTX-M-15_, *bla*_TEM-1B_, *bla*_OXA-232_, *bla*_SHV-1_ | *aac*(6')-Ib, *aad*A2, *rmt*F | *qnr*S1, *par*C(S80I) | *fos*A6 |
| KPCRETH15 | *K. pneumoniae* | SAMN07203030 | *bla*_TEM-1B_, *bla*_OXA-232_, *bla*_SHV-1_ | *aad*A2, *rmt*F | *qnr*S1, *par*C(S80I) | *fos*A6, *Uhp*T |
| KPCRETH16 | *K. pneumoniae* | SAMN07203029 | *bla*_TEM-1B_, *bla*_OXA-232_, *bla*_SHV-1_ | *aad*A2, *rmt*F | *qnr*S1, *par*C(S80I) | *Uhp*T |
| KPCRETH17 | *E. hormaechei* | SAMN07450559 | *bla*_CTX-M-15_, *bla*_TEM-1B_, *bla*_OXA-10_, *bla*_ACT-17_, *bla*_IMP-14_ | *aph*(3'')-Ib, *aac*(6')-Ib3, *aph*(6)-Id | *par*C(S80I), *gyr*A(E88A) | *Uhp*T |
| KPCRETH18 | *K. pneumoniae* | SAMN07450567 | *bla*_CTX-M-15_, *bla*_NDM-1_, *bla*_OXA-1_, *bla*_SHV-1_ | *aac*(6')-Ib-cr, *aph*(3'')-Ib, *aph*(6)-Id | *qnr*B1, *par*C(S80I) | *fos*A6, *Uhp*T |
| KPCRETH19 | *K. pneumoniae* | SAMN07450566 | *bla*_CTX-M-15_, *bla*_NDM-1_, *bla*_OXA-1_, *bla*_SHV-1_ | *aac*(6')-Ib-cr, *aph*(3'')-Ib, *aph*(6)-Id | *qnr*B1, *par*C(S80I) | *fos*A6 |
| KPCRETH20 | *K. pneumoniae* | SAMN07450565 | *bla*_CTX-M-15_, *bla*_NDM-1_, *bla*_OXA-1_, *bla*_SHV-1_ | *aac*(6')-Ib-cr, *aph*(3'')-Ib, *aph*(6)-Id | *qnr*B1, *par*C(S80I) | *fos*A6, *Uhp*T |
| KPCRETH21 | *K. pneumoniae* | SAMN07450564 | *bla*_CTX-M-15_, *bla*_TEM-1B_, *bla*_OXA-232_, *bla*_SHV-1_ | *aad*A2, *rmt*F | *qnr*S1, *par*C(S80I) | *fos*A6, *Uhp*T |
| KPCRETH22 | *K. pneumoniae* | SAMN07450563 | *bla*_CTX-M-15_, *bla*_TEM-1A_, *bla*_NDM-1_, *bla*_OXA-232_, *bla*_SHV-1_ | *aad*A2 | *gyr*A(D95N) | *fos*A6, *Uhp*T |
| KPCRETH23 | *K. pneumoniae* | SAMN07450562 | *bla*_CTX-M-15_, *bla*_TEM-1A_, *bla*_NDM-1_, *bla*_OXA-232_, *bla*_SHV-1_ | *aad*A2 | *gyr*A(D95N) | *Uhp*T |
| KPCRETH24 | *K. pneumoniae* | SAMN07450561 | *bla*_CTX-M-15_, *bla*_TEM-1A_, *bla*_NDM-1_, *bla*_OXA-232_, *bla*_SHV-1_ | aadA2 | *gyr*A(D95N) | *fos*A6, *Uhp*T |
| KPCRETH25 | *K. pneumoniae* | SAMN07450560 | *bla*_CTX-M-15_, *bla*_TEM-1A_, *bla*_OXA-232_, *bla*_OXA-9_, *bla*_SHV-1_ | *aac*(6')-Ib | *gyr*A(D95N) | *fos*A6, *Uhp*T |
| KPCRETH26 | *K. pneumoniae* | SAMN07450558 | *bla*_CTX-M-15_, *bla*_TEM-1B_, *bla*_OXA-232_, *bla*_SHV-1_ | *aad*A2, *rmt*F | *qnr*S1, *par*C(S80I) | *fos*A6, *Uhp*T |
| KPCRETH27 | *K. pneumoniae* | SAMN07450557 | *bla*_CTX-M-15_, *bla*_TEM-1B_, *bla*_OXA-232_, *bla*_SHV-1_ | *aad*A2, *rmt*F | *qnr*S1, *par*C(S80I) | *fos*A6, *Uhp*T |
| KPCRETH28 | *K. pneumoniae* | SAMN07450556 | *bla*_CTX-M-15_, *bla*_TEM-1A_, *bla*_NDM-1_, *bla*_OXA-232_, *bla*_OXA-9_, *bla*_SHV-1_ | *aad*A1, *aad*A2, *aac*(6')-Ib | *gyr*A(D95N) | *fos*A6, *Uhp*T |
| KPCRETH29 | *K. pneumoniae* | SAMN07450555 | *bla*_CTX-M-15_, *bla*_NDM-1_, *bla*_OXA-232_, *bla*_OXA-1_, *bla*_SHV-28_ | *aad*A2, *aac*(6')-Ib-cr, *aph*(3')-VI, *arm*A | *qnr*B1, *gyr*A(D105G), *par*C(E84G) | *fos*A6, *Uhp*T |
| KPCRETH30 | *K. pneumoniae* | SAMN07450554 | *bla*_CTX-M-15_, *bla*_NDM-1_, *bla*_OXA-232_, *bla*_OXA-1_, *bla*_SHV-28_ | *aad*A2, *aac*(6')-Ib-cr, *aph*(3')-VI, *arm*A | *qnr*B1, *gyr*A(D105G), *par*C(E84G) | *fos*A6, *Uhp*T |
| KPCRETH31 | *K. pneumoniae* | SAMN07450553 | *bla*_CTX-M-15_, *bla*_TEM-1B_, *bla*_OXA-232_, *bla*_SHV-1_ | *aad*A2, *rmt*F | *qnr*S1, *par*C(S80I) | *Uhp*T |
| KPCRETH32 | *K. pneumoniae* | SAMN07450552 | *bla*_CTX-M-15_, *bla*_TEM-1B_, *bla*_OXA-232_, *bla*_SHV-1_ | *aad*A2, *rmt*F | *qnr*S1, *par*C(S80I) | *fos*A6, *Uhp*T |
| KPCRETH33 | *K. pneumoniae* | SAMN07450581 | *bla*_CTX-M-15_, *bla*_TEM-1B_, *bla*_OXA-232_, *bla*_SHV-1_ | *aad*A2, *rmt*F | *qnr*S1, *par*C(S80I) | *fos*A6, *Uhp*T |
| KPCTRPRTH01 | *K. pneumoniae* | SAMN07450577 | *bla*_CTX-M-15_, *bla*_TEM-1A_, *bla*_NDM-1_, *bla*_OXA-232_, *bla*_OXA-9_, *bla*_SHV-1_ | *aad*A1, *aad*A2, *aac*(6')-Ib | *gyr*A(D95N) | *fos*A6 |
| KPCTRPRTH02 | *K. pneumoniae* | SAMN07450622 | *bla*_CTX-M-15_, *bla*_TEM-1C_, *bla*_NDM-1_, *bla*_OXA-232_, *bla*_SHV-1_ | *aad*A2, *aac*(6')-Ib-cr | *gyr*A(D95N) | *Uhp*T |
| KPCTRPRTH03 | *K. pneumoniae* | SAMN07450621 | *bla*_CTX-M-15_, *bla*_TEM-1B_, *bla*_OXA-232_, *bla*_SHV-1_ | *aad*A2, *rmt*F | *qnr*S1, *par*C(S80I) | *fos*A6, *Uhp*T |
| KPCTRPRTH04 | *K. pneumoniae* | SAMN07450620 | *bla*_CTX-M-15_, *bla*_NDM-1_, *bla*_OXA-232_, *bla*_SHV-1_ | *aad*A2 | *gyr*A(D95N) | *Uhp*T |
| KPCTRPRTH05 | *K. pneumoniae* | SAMN07450619 | *bla*_CTX-M-15_, *bla*_TEM-1A_, *bla*_NDM-1_, *bla*_OXA-232_, *bla*_OXA-9_, *bla*_SHV-1_ | *aad*A1, *aad*A2, aac(6')-Ib | *gyr*A(D95N) | *fos*A6, *Uhp*T |
